# Regional abnormality of functional connectivity is associated with clinical manifestations in individuals with intractable focal epilepsy

**DOI:** 10.1038/s41598-021-81207-6

**Published:** 2021-01-15

**Authors:** Yasuo Nakai, Hiroki Nishibayashi, Tomohiro Donishi, Masaki Terada, Naoyuki Nakao, Yoshiki Kaneoke

**Affiliations:** 1 Department of Neurological Surgery, Wakayama Medical University, 811-1 Kimiidera, Wakayama, 641-8509 Japan; 2 Department of System Neurophysiology, Wakayama Medical University, 811-1 Kimiidera, Wakayama, 641-8509 Japan; 3 Wakayama-Minami Radiology Clinic, 870-2 Kimiidera, Wakayama, 641-0012 Japan

**Keywords:** Epilepsy, Network models

## Abstract

We explored regional functional connectivity alterations in intractable focal epilepsy brains using resting-state functional MRI. Distributions of the network parameters (corresponding to degree and eigenvector centrality) measured at each brain region for all 25 patients were significantly different from age- and sex-matched control data that were estimated by a healthy control dataset (n = 582, 18–84 years old). The number of abnormal regions whose parameters exceeded the mean + 2 SD of age- and sex-matched data for each patient were associated with various clinical parameters such as the duration of illness and seizure severity. Furthermore, abnormal regions for each patient tended to have functional connections with each other (mean ± SD = 58.6 ± 20.2%), the magnitude of which was negatively related to the quality of life. The abnormal regions distributed within the default mode network with significantly higher probability (p < 0.05) in 7 of 25 patients. We consider that the detection of abnormal regions by functional connectivity analysis using a large number of control datasets is useful for the numerical assessment of each patient’s clinical conditions, although further study is necessary to elucidate etiology-specific abnormalities.

## Introduction

Approximately 25% of epilepsy patients are medically intractable^1^. For these patients, various types of brain surgeries have been performed to improve their quality of life (QOL) by reducing seizure frequency^2^. However, long-term outcomes, such as the seizure freedom rate, have been stagnating, and treatment of intractable focal epilepsy is still challenging^3^. Previous studies showed that even for temporal lobe epilepsy (TLE) with mesial temporal lobe sclerosis, the seizure freedom rate was 53–84% after anteromesial temporal lobe resections. Similarly, the seizure freedom rate for patients with localized neocortical epilepsy was 36–76%^4^. These data suggest that the epileptogenic zone extends outsize the local brain lesions for some patients, and these epileptogenic zones could not be detected by conventional methodologies such as scalp or intracranial electroencephalography (EEG), structural magnetic resonance imaging (MRI), magnetoencephalography, and positron emission tomography. It is often assumed that the epileptogenic zone extends by way of the brain network; for example, mirror focus arises in the contralateral hemisphere in temporal lobe epilepsy in its clinical course^5^. The epileptogenic zone extends to the whole brain and the effect of brain surgery worsen over time^6^.

Recently, resting-state functional MRI (rs-fMRI) studies have been used to elucidate normal and various abnormal brain functional networks^7–9^ and many studies have shown that epilepsy patients have abnormal brain networks as measured by rs-fMRI^10–13^. However, the majority of studies performed group analyses that did not focus on individual epilepsy network alterations. If each individual brain network could be assessed before surgery, a better surgical outcome might be achieved. We consider that focal epilepsy has a common pathophysiological brain functional abnormality even if its epileptic focus is varied. Corresponding to this idea, some anti-epileptic drugs such as carbamazepine are effective for focal epilepsy but not for generalized epilepsy^14^. Thus, we investigated whether brain network abnormalities could be assessed in individuals with various types of intractable focal epilepsies by comparing a large number of brain network data from healthy controls. Brain network structure could vary with various causes such as age^15,16^, sex^17^, and intelligence^18,19^, so a large amount of control data is necessary to determine the normal range and to assess each individual brain network structure.

Centrality analysis using rs-fMRI has detected alterations in the network in several epilepsy types^10,20,21^, and initial observations suggested a network disruption in epilepsy and disease progression as well as postsurgical seizure outcome, which emphasizes that graph theoretical analysis may lend markers for disease staging and prognostics. In this present study, we employed normalized α-centrality (nAC) on rs-fMRI^22,23^ to quantify the brain regional network organization to compare different centralities. The nAC measures the strength of a node in a network from the local viewpoint to the global viewpoint by changing the parameter (that is, α) from zero to 1/λ; thereby, λ is the maximum eigenvalue of the adjacency matrix of the network. We calculated nAC values at each brain region with two different α values (0 and 1/λ) to determine the regional abnormality. Locally connected nodes were reflected by nAC at α = 0 (nAC_0_), which corresponds to degree centrality. Conversely, globally connected nodes were reflected by nAC at α = 1/λ (nAC_1_), which correspond to eigenvector centrality. nAC_0_ reflects how many regions are connected to a given region, whereas a large nAC_1_ reflects how strongly the connectivity of a given region contributes to the whole network.

The aim of this study was to elucidate abnormal brain regions using network properties that are specific to each individual with intractable focal epilepsy by comparison with a relatively large number of control data. If it was possible, we assessed the relationship between these regional abnormalities and clinical parameters such as the QOL or seizure severity of patients.

## Results

### Patient characteristics

Twenty-five patients with intractable (or drug-resistant) focal epilepsy (9 females and 16 males, age ranged from 16 to 70 years old, mean ± SD: 39.1 ± 12.3 years) participated in this study. The duration of illness ranged from 1 to 42 years with a mean of 19.8 years; the number of antiepileptic drugs ranged from 1 to 4 with a mean of 2.8. Three patients (cases 1, 12, and 13) underwent epilepsy surgery monitoring with electrocorticography (ECoG) after the MRI study. Twelve patients (cases 1–12) had structural lesions (5 cortical malformations, 3 tumourous lesions, 2 hippocampal atrophy, 1 amygdala lesion, and 1 hippocampal lesion). The demographic data for the patients including age at rs-fMRI, sex, epilepsy onset, duration of epilepsy, etiology, seizure type, EEG, and MRI findings are summarized in Supplementary Table S1. We divided the patients into three groups based on the clinical data as follows: lobar type of temporal lobe (TLE type, n = 10), lobar type of extra-temporal lobe (focal type, n = 5), and multilobar type (n = 10)^24^.

### Network characteristics

The number of nodes was 388 because the cerebral gray matters were divided into 368 regions using Atlas of Intrinsic Connectivity of Homotopic Areas (AICHA)^25^ and the cerebellar gray matters were divided into 20 regions using Automated Anatomical Labeling atlas (AAL)^26^ for 20 regions in the cerebellum (Supplementary Table S2). However, the number of edges varied with individuals as the functional connectivity strength measured by Pearson’s correlation coefficient between two regions was binarized at Z > 1.96 to make an adjacency matrix for each subject (see “Materials and methods”). The mean number of edges was 6224 ± 1180 for the patients and 6845 ± 1201 for the controls. The analysis of covariance revealed that the number of edges was significantly different between the controls and the epilepsy patients with age as a nuisance covariate (df = 1, F = 7.19, p = 0.0075) and there was a significant interaction between group and age (df = 1, F = 6.97, p = 0.0085) (Supplementary Fig. S1). The number of edges was not related to the clinical parameters of the patients, such as duration of illness (p > 0.05, Spearman’s partial correlation analysis excluding the effect of age), the number of anti-epileptic drugs (AEDs), the Quality of Life in the Epilepsy Inventory (QOLIE-31) and Liverpool Seizure Severity Scale (LSSS) (p > 0.05, Spearman’s correlation analysis).

### Distributions of nAC_0_ and nAC_1_

**Figure 1:**
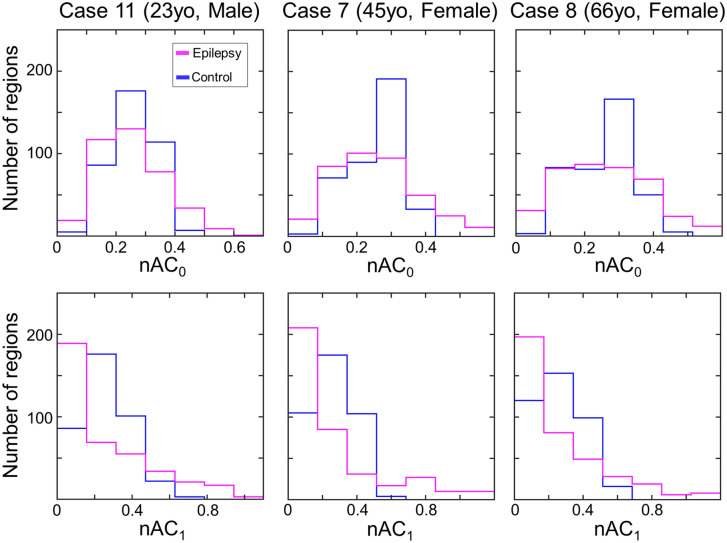
Distributions of nAC values for the patients were different from those for controls. The distributions of nAC_0_ and nAC_1_ are shown for three representative cases. The number of regions with lower (< 0.2) and higher (> 0.5) values were larger in the epilepsy patients than those for healthy controls, which was the case for all the patients (see Supplementary Fig. S1).

The frequency distributions of the nAC values for each subject are calculated, and the representative data are shown in Fig. 1 (all the data are shown in Supplementary Figs. S2, S3). We found that the number of regions with lower nAC values (< 0.2) and higher nAC values (> 0.5) were larger than those for the controls for all the patients. Chi-square tests revealed that distributions of nAC_0_ and nAC_1_ for each patient were significantly difference from those for age- and sex-matched controls (p < 0.005). Spatial distributions of these values for the healthy controls (mean values for the 20, 40, and 60 years old) are shown in Supplementary Fig. S4.

### Definition of mathematical measure

**Figure 2:**
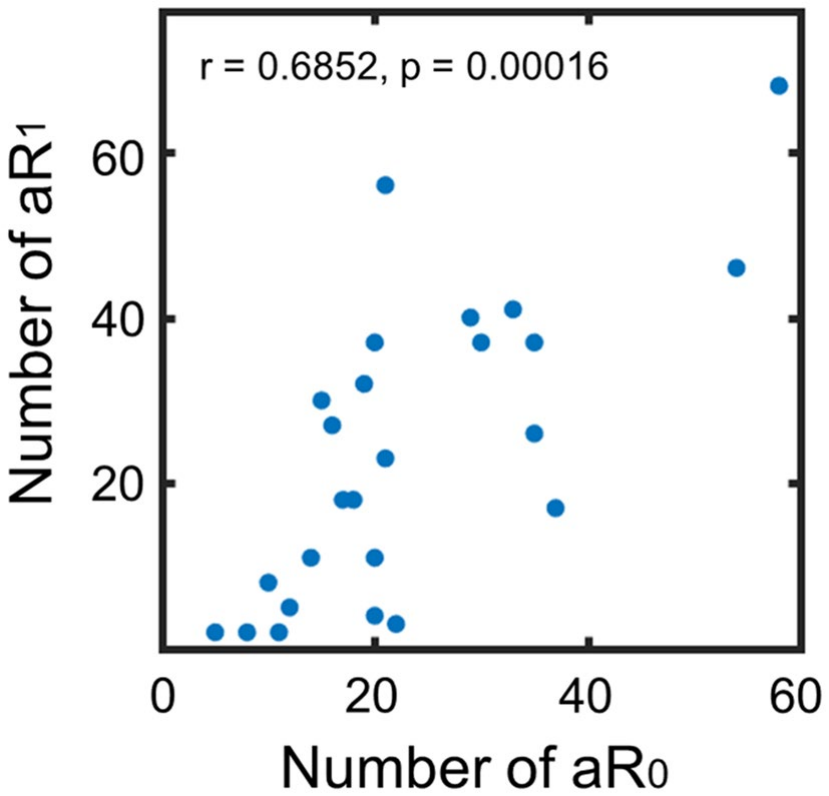
The number of aR_0_ was related to that of aR_1_. The number of aR_0_ had a positive correlation with the number of aR_1_ (r = 0.6852, p = 0.00016, Spearman’s method).

We defined abnormal regions (aR_0_ and aR_1_) whose nAC_0_ and nAC_1_, respectively, were greater than the mean + 2 SD of the age- and sex-matched values. The relative frequency distribution of the aR_0_ and aR_1_ for both healthy controls and patients are shown in Supplementary Figure S5. As shown, the distributions were not normal. Chi-square tests revealed that distributions for the patients were significantly different from control data (χ^2^ = 147, p < 0.00001 for aR_0_; χ^2^ = 23.04, p = 0.00078 for aR_1_). The median number of aR_0_ and aR_1_ for the patients were 19.5 and 18.0, respectively. The number of aR_0_ was significantly higher than that for the controls (median = 10.5) (z = 3.5901, p = 0.00033, Wilcoxon rank sum test), whereas the number of aR_1_ was not significantly different from that for the controls (median = 14) (z = 0.7833, p > 0.05). However, the number of aR_1_ for the patients was significantly related to the number of aR_0_ (r = 0.6852, p = 0.00016, Spearman’s correlation analysis) as shown in Fig. 2.

We employed the kappa statistic^27^ to check how much normal and abnormal regions defined by nAC_0_ showed agreement with those defined by nAC_1_ (Supplementary Figure S6). The mean ± SD of the values was 0.518 ± 0.162, which indicates that the agreement was moderate.

### Relationship between abnormal regions and clinical parameters

**Figure 3:**
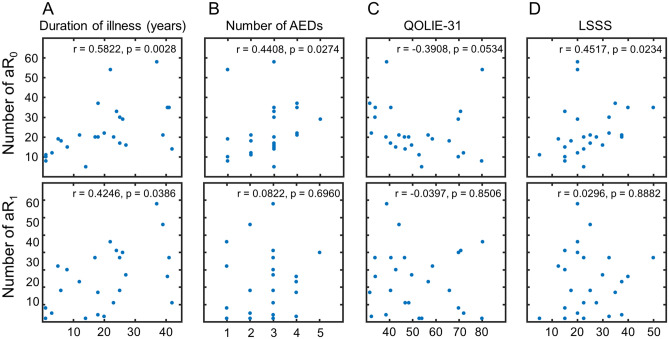
Number of abnormal regions were associated with various clinical parameters. Spearman’s partial correlation analysis with age as nuisance covariate showed that the numbers of aR_0_ and aR_1_ were related to the duration of illness (r = 0.5822, p = 0.0028; r = 0.42646, p = 0.0386, respectively) (**A**). The number of aR_0_ also was related to the number of AEDs (**B**) and LSSS (**D**). *AED* anti-epileptic drug, *QOLIE* Quality of life in Epilepsy Inventory, *LSSS* Liverpool Seizure Severity Scale.

The numbers of aR_0_ and aR_1_ were related to the duration of illness as revealed by Spearman’s partial correlation analysis excluding the effect of age (r = 0.5822, p = 0.0028; r = 0.4246, p = 0.0386, respectively). Partial correlation analysis was used because the patient’s age was related to the duration of illness (r = 0.5067, p = 0.0098, Spearman’s correlation analysis). Further, the number of aR_0_ was related to the number of AEDs and LSSS (r = 0.4408, p = 0.0274; r = 0.4517, p = 0.0234, respectively, Spearman’s correlation analysis). Figure 3 shows the scatter plots of the data. Both the number of abnormal regions (aR_0_ and aR_1_) for the patients with impaired awareness was not significantly different from those of the patients without impaired awareness (p > 0.05, *t* test). There was also no significant difference between the number of abnormal regions for the patients with structural lesions and those without lesions (p > 0.05, *t* test).

**Figure 4:**
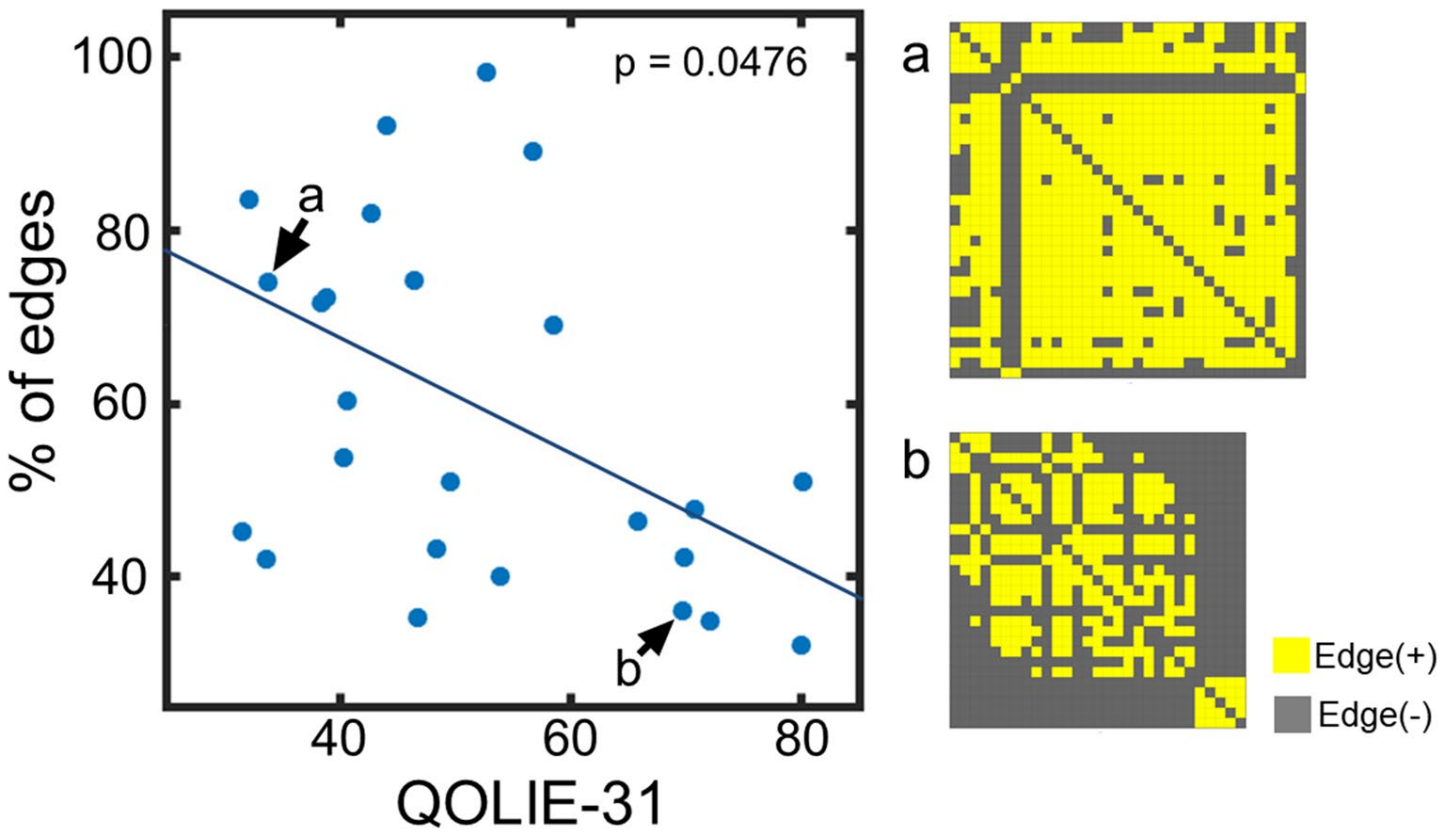
Functional connectivity within aR_0_ was negatively related to QOLIE-31. As the percentage of edges within the abnormal regions (aR_0_) increased, the patient’s QOL was worse (that is, QOLIE-31 was low). The right of the figure shows the visual matrices indicating the edges between abnormal regions (aR_0_) corresponding to the plots (**a**,**b**) in the graph at the right side of the figure.

The mean percentage of connections (number of edges among abnormal regions divided by the number of possible edges, that is *C*(*n*, 2), where *n* is the number of abnormal regions and *C* is the combinations formula) was 58.6% (ranges from 32.1 to 98.2%). Visual matrices of representative cases are shown in Fig. 4, and all the data are shown in Supplementary Figure S7. The percentage of connections for aR_0_ was related to QOLIE-31 as revealed by Spearman’s correlation (r = − 0.4015, p = 0.0476) (Fig. 4).

### Relationship between abnormal regions and default mode network

**Figure 5:**
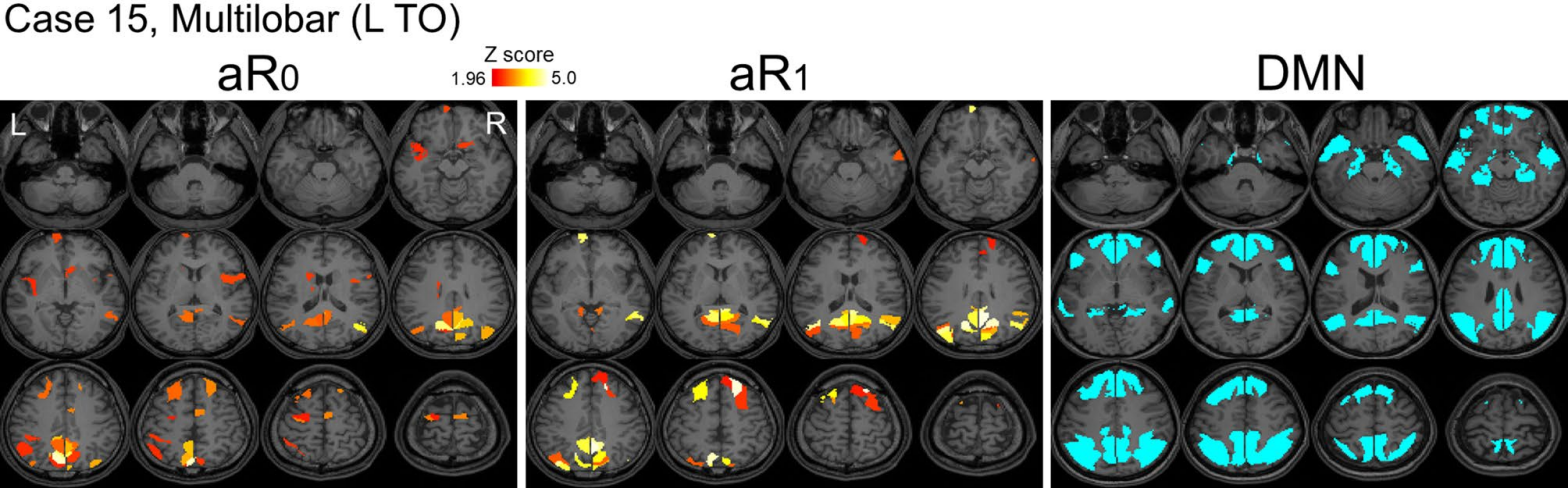
Abnormal regions were found with the default mode network for some patients. The spatial distribution of abnormal aR_0_ and aR_1_ of representative case (case 15) and the regions of the default mode network (DMN) are shown. This patient had 19 of 33 aR_0_ regions (57.6%) and 26 of 41 aR_1_ regions (63.4%) included in the DMN, which is significantly higher (p < 0.05, Chi-square test) than the expected value (25.8%). *L TO* left temporo-occipital lobe.

At least one of aR_0_ or aR_1_ for 23 (92%) and 18 (72%) patients was within the DMN (see Supplementary Table S2 for the regions in DMN). For 6 and 7 patients, the percentage of abnormal regions (aR_0_ or aR_1_) within the DMN was significantly higher than the value expected from the number of the DMN regions (n = 100 of 388 regions, that is 25.8%), respectively (p < 0.05, Chi-square test). For five patients, the percentages of both aR_0_ and aR_1_ within the DMN were significantly larger than the expected value (p < 0.05, Chi-square test). Figure 5 shows the spatial distribution of aR_0_ and aR_1_ of a representative case and the regions of DMN. Supplementary Table S3 shows the percentage of abnormal regions within the DMN for all the patients. The number of abnormal regions within the DMN was not related to the clinical parameters (p > 0.05, Spearman’s test).

### Relationship between abnormal regions and estimated epileptic focus

**Figure 6:**
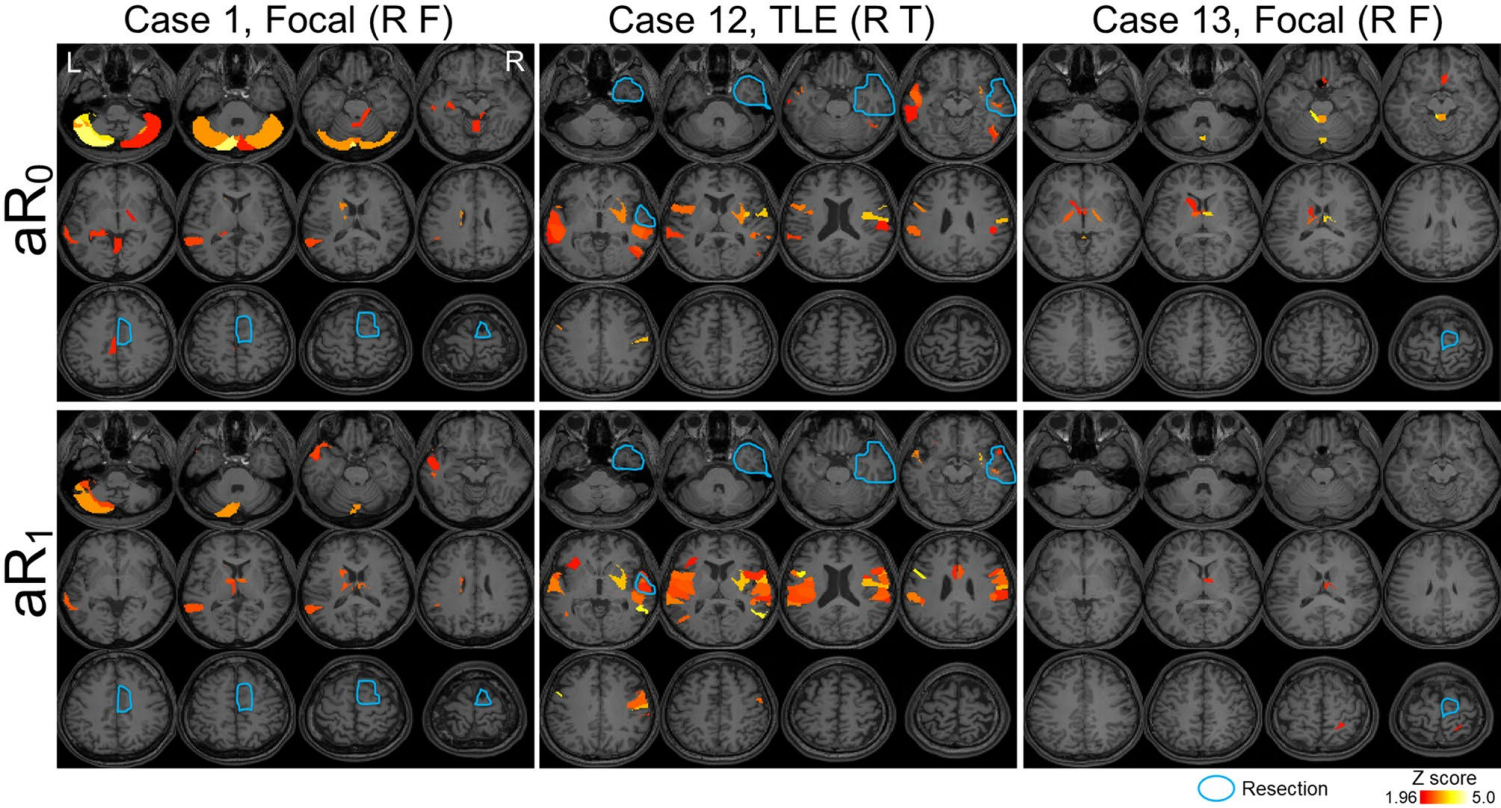
Surgically resected areas did not always include abnormal regions. The spatial distributions of aR_0_ and aR_1_ and resection areas for the three surgical cases are shown. Resection areas were determined by ECoG during the surgery. In case 12, the area included the abnormal regions (both aR_0_ and aR_1_), but the number was not larger than the value expected by chance.

Epileptic focus could be reliably estimated by ECoG before resection surgery as epileptogenic zone. Thus, we checked the relationship between distribution of abnormal regions and epileptic focus estimated by ECoG. Three patients underwent resection surgery after the MRI study and all of them have been seizure-free. The resection areas overlapped with aR_0_ and aR_1_ in one patient with temporal lobe epilepsy (case 12), whereas the other resection areas did not include abnormal regions for neocortical epilepsy patients (case 1 and 13). In case 12, the seizure onset zone based on ECoG findings included not only the right mesial temporal area but also the right insula and lateral temporal area. Figure 6 shows the resection areas and the abnormal regions for the three patients. The number of abnormal regions did not exceed the number expected by the total number of abnormal regions when the abnormal regions distributed randomly (p > 0.05, Chi-square test).

## Discussion

We investigated brain regional functional connectivity abnormalities in 25 patients with intractable focal epilepsy using rs-fMRI data. Even though the type of epilepsy was heterogeneous, we could elucidate aberrant brain regions for each patient by the measurement of nAC values comparing a large number (n = 582) of healthy control data. The distributions of nAC_0_ and nAC_1_ showed that the number of regions with the lowest and the highest values increased for all the patients compared to the age- and sex-matched control data (Fig. 1). Although the number of aR_1_ for the patients is not significantly higher than that for the controls, there was a positive correlation between aR_0_ and aR_1_ and the distributions of the number of aR_0_ and aR_1_ for the patients were significantly different from the controls (Fig. 2; Supplementary Fig. S5). The number of abnormal regions was related to the clinical parameters, such as the duration of illness, number of AEDs, and LSSS (Fig. 3). The locations of the abnormal regions were around the estimated epileptic focus in 64% of patients. The percentage of edges between abnormal regions (aR_0_) was negatively related to the QOLIE-31 (Fig. 4). These results suggest that abnormal regions were detected not by chance but are related to the pathophysiology of epilepsy.

Our network analysis using normalized α-centrality suggests the existence of inter-ictal network abnormalities that are common to focal epilepsy irrespective of the causes, which corresponds to the recent meta-analysis of focal epilepsy network studies^28^. These researchers observed a significant increase in path length and clustering coefficient for focal epilepsy patients compared to healthy controls, and the values were not significantly different among temporal lobe, extra-temporal, and brain tumor patients. We found that the distributions of nAC_0_ and nAC_1_ for each patient were significantly different from those for age- and sex-matched controls (Fig. 1; Supplementary Figs. S2, S3) even though the number of aR_1_ for the patients is not significantly higher than that for the controls, and the number of edges was significantly different from that for age- and sex-matched control values. These abnormalities could be related to epilepsy symptoms, as previously discussed^29–34^. These studies used graph theoretical analysis with parameters such as path length and clustering coefficient to elucidate aberrant network structures that are associated with epilepsy in the brain. To compare two graphs using these network parameters, the number of nodes and edges must be equivalent. For the brain network analysis, whether the two regions are connected commonly by the correlation coefficient should be verified. The threshold for constructing an adjacency matrix must be varied to obtain the same number of edges for all the subjects. In this study, we preferred to use the same threshold for the adjacency matrix even though it produced a different number of edges among the subjects, and a detailed network structure comparison could not be possible. The merit of using the same threshold is that the existence of an edge clearly indicates that the functional connectivity strength was larger than the same threshold (Z value of correlation coefficient), which is definitely determined statistically. Thus, the number of edges in a given region means how many regions a given region is functionally connected with a determined strength (threshold), which can be directly compared with other brain regions^23^. Although our previous studies^35,36^ showed the weighted degree centrality is also useful for other diseases, we did not use it in this study. The value of weighted degree centrality reflects both number of edges and magnitude of the cross-correlation coefficient which makes the interpretation difficult when the patients’ data was compared with normal range.

Even though the number of edges for the patients was smaller than those for the controls (Supplementary Fig. S1 to show the number of edges), we found that the number of regions with larger nAC values for all the patients were larger than those for controls (Fig. 1, Supplementary Figs. S2, S3). Thus, we determined the brain regions (aR_0_ and aR_1_) that had abnormally larger nAC_0_ and nAC_1_ values in epilepsy patients compared to the age- and sex-matched controls. We consider that both aR_0_ and aR_1_ are related to the pathophysiology of epilepsy for the following reasons: the frequency distributions of the number of abnormal regions for the patients were significantly different from those for the healthy controls (Supplementary Fig. S5) and the number of aR_0_ for the patients were significantly different from that for the controls. We also found a positive correlation between the number of aR_0_ and aR_1_ (Fig. 2). Further, the number of aR_0_ and aR_1_ were related to the various clinical parameters (Fig. 3). Abnormal regions (aR_0_) had edges between each other (mean ± SD = 58.6 ± 20.2%), and the percentage of edges among the abnormal regions was negatively related to QOLIE-31 (Fig. 4). Because the number of aR_1_ was only related to the duration of illness, aR_0_ might be useful parameter more than aR_1_ to assess epilepsy status. It is possible though that aR_1_ might be related to the other aspects of epilepsy status since the spatial distribution of aR_1_ was *moderately* similar to that of aR_0_.

Although it is not certain if seizures cause brain network changes or epileptic networks induce seizures^28^, an increase in the number of abnormal regions might be related to the spatial extension of the abnormal network as the values were related to the duration of illness (Fig. 3A) and the patients’ seizure severity (Fig. 3B,D). The duration of illness has been shown to reduce the seizure-free rate after surgery^37,38^, and functional connectivity alterations in the epilepsy brain have been observed with various parameters^31,39,40^. Haneef et al. also showed that the network structure changes with duration of illness for the patients with temporal lobe epilepsy. They observed that the networks became homogenized with the duration. The result does not necessarily contradict with ours, because existence of abnormal regions which have larger centralities could simplify the whole brain network structures. If majority of the rest of nodes have a few numbers of edges within a restricted range, connectivity diversity of the network could be lower than the controls. However, we cannot directly compare our results with theirs, because the number of edges were varied with subjects in our study.

For seven patients, the number of abnormal regions found in the default mode network was significantly larger than the chance level. Although we could not find any specific characteristics common to these seven patients, further study might elucidate clinical significance of abnormal regions within the default mode network (DMN)^25^, because the core network nodes of the DMN have been highly involved in the network of patients with temporal lobe epilepsy^41^. However, aR_0_ and aR_1_ were not associated with the estimated epileptic focus based on three surgical cases with ECoG in our study. This finding might not be odd as our MRI measurements were performed in the inter-ictal period of the patients and the regional blood flow of the epileptic focus has been shown to be reduced^42^, which means that the neural activity around the focus is low, and might reduce the functional connectivity with other regions. In the previous rs-fMRI study, temporal lobe epilepsy had disrupted connectivity of the ipsilateral mesiotemporal lobe, with contralateral compensatory reorganization and striking reconfigurations of large-scale networks^43^, which may support our findings. The neural activity of the epileptic focus might be independent of other brain regions in the resting state, so functional connectivity did not increase. We observed that the spatial distribution of aR_0_ was similar to that of aR_1_ for each patient, but some abnormal regions were detected only in aR_0_ or aR_1_ (Supplementary Fig. S6). The mean ± SD of the kappa statistic among the patients was 0.518 ± 0.162, which indicates moderate agreement. As nAC_0_ is related to local connectivity and nAC_1_ is related to global network connectivity^22^, comparison of the spatial distribution of aR_0_ with that of aR_1_ might reveal the pathological significance of these regions.

It is reasonable that the control subjects also had a number of abnormal regions because these were determined by the mean + 2 SD of age- and sex-matched control nAC value for each region. This means that there are 2.5% of controls whose nAC value at a region exceeded the mean + 2 SD. Possibly, spatial distributions of the *abnormal* regions for the controls would be different from those for the patients. We did not analyze the possibility because our objective is to assess each patient’s epilepsy status using the abnormal regions and not to make a differential diagnosis of epilepsy patients by the detection of abnormal regions.

There are some limitations in this study. First, according to the ILAE 2017 classification^44^, “Aware or Impaired awareness” and “Structural or not” are the important factors for classifying seizure type of focal onset and etiology, respectively. These factors did not affect the number of abnormal regions (p > 0.05, *t* test) possibly due to the low number of patients’ data and various etiologies (see Supplementary Table S1). Detailed studies with homogeneous patient groups, such as temporal lobe epilepsy, can detect abnormalities that are specific to the epilepsy group. Second, our MRI acquisition was performed during the inter-ictal state for the patients as in the previous MRI studies for epilepsy. Brain networks in the patients may vary depending on the alertness states (from the fully awake stage to the deep sleep stage). Even in awake conditions, the existence and frequency of epileptic discharge could affect the brain network^45^. Third, the effect of medication with AED could not be explored in this study as all of our patients had AED medication. Previous studies have shown that AED can reduce the functional connectivity of the epileptic brain^31,46^. Thus, our findings might represent underestimated abnormalities in epileptic brain networks. Furthermore, it is well-known that sex hormones affect brain functions^47^ and a distinct difference in epilepsy symptoms exists between men and women^48–50^. The distributions of abnormal regions could vary with the menstrual cycle in female patients.

In conclusion, we elucidated the abnormal brain regions in intractable focal epilepsy using functional connectivity analysis by comparing a large number of healthy control data. The number of abnormal regions (aR_0_ and aR_1_) might be a useful parameter for the numerical assessment of the effect of AED treatment and indication of surgical treatment for each patient as the value was related to the duration of illness and clinical parameters such as the QOLIE-31 and LSSS.

## Materials and methods

### Participants

This study was approved by the ethical committee at Wakayama Medical University (#1182 and 2664). All participants gave written informed consent prior to the study, which was carried out in accordance with the latest version of the Declaration of Helsinki. For the participants under the age of 18 years, informed consent from parent/legal guardian was obtained. Twenty-five patients with intractable, or drug-resistant, focal epilepsy participated in this study. Based on the latest International League Against Epilepsy operational classifications, all participants were evaluated by expert epileptologists between December 2012 and June 2014. The demographic data for the patients including age at rs-fMRI, sex, epilepsy onset, duration of epilepsy, etiology, seizure type^44^, EEG, and MRI findings are summarized in Supplementary Table S1. The epileptic focus for each patient was estimated by EEG and structural MRI findings and divided into three groups: lobar type of temporal lobe (TLE type, n = 10), lobar type of extra-temporal lobe (focal type, n = 5), and multilobar type (n = 10)^24^.

We used rs-fMRI data from 582 healthy subjects (222 males, mean ± SD: 34.2 ± 20.7 years, range 18–83 years; 360 females, mean ± SD: 36.8 ± 19.9 years, range 18–84 years) who were recruited by the Department of System Neurophysiology and School of Health and Nursing Science at Wakayama Medical University.

### Image acquisition

We acquired structural and functional images on a 3 T MRI (PHILIPS, The Netherlands) using a 32CH-channel head coil (SENSE-Head-Netherlands). High resolution three-dimensional T1-weighted anatomical images were collected with the following parameters: TR = 6.9 ms, TE = 3.3 ms, FOV = 256 mm, matrix scan = 256, slice thickness = 1.0 mm, and flip angle = 10°. Functional data were acquired using a gradient-echo echo-planar pulse sequence sensitive to BOLD contrast^51^ with the following parameters: TR = 3000 ms, TE = 30 ms, FOV = 192 mm, Matrix scan = 64, slice thickness = 3.0 mm, and flip angle = 80°. Three runs, each with 105 volumes, were administered to each subject. During the acquisition, the subjects were instructed to stay awake with their eyes closed.

### MRI data analysis

Preprocessing of functional MRI data was conducted using SPM8 and in-house software that was developed with MATLAB (The MathWorks, Inc., USA). The first 3 volumes of each fMRI acquisition run were discarded to enable T1-equilibration effects for a total of 102 consecutive volumes per session. Rigid body translation and rotation were used to correct head motion and spatial normalization according to the International Consortium for Brain Mapping Echo Planar Imaging template. Each image was resampled to 2-mm isotropic voxels and spatially smoothed using an 8-mm full width at half maximum Gaussian kernel. Similar normalized and resampled structural images were employed to extract time series data for the gray matter (GM), which were applied to reduce non-physiological noise in the time series of BOLD signals. GM mask images were generated using SPM8 with a probability threshold of 90% as an initial mask. Exclusion of the signals unrelated to brain function, such as brain tissue fluctuations due to head motion, cardiac activity, and respiration, was performed using CompCor^52^. Slice time adjustment was performed using spline interpolation by aligning the bottom of the slices, which produced in 101 volumes for each session. Temporal (band-pass) filtering (ranges from 0.01 to 0.1 Hz) removed constant offset and linear trends over each run. The preprocessed images for each session were concatenated signal four-dimensional (time and 3 spatial data) images, and thus, the data from the 3 sessions for each subject were utilized for the following analysis. Sessions with large motion such as a rotation of ≥ 0.02 radians or a translation of ≥ 2 mm, were excluded. For computational efficiency and anatomical variance among the subjects, voxels within the GM were down-sampled to 6-mm isotropic voxels to make the number of gray matter voxel to be the same for the normal data as our previous study^23^.

### Functional connectivity analysis

The brain GM was divided into 388 regions using AIHCA^25^ for the cerebrum (368 regions) and AAL^26^ for the cerebellum (20 regions) (see Supplementary Table S2). BOLD signals from GM were averaged at each brain regions to calculate the strength of the functional connectivity between one region and all the other regions in the brain using Pearson’s correlation coefficient (r). Each value (r) was converted to a t value using the effective sample size calculated with the autocorrelation coefficient in each region^53^. The t value was then converted to the Z value. For each subject, Z values at each voxel for 3 sessions were averaged. The adjacency matrix for each subject was created from the Z value matrix using the threshold Z > 1.96 (p < 0.05). We employed nAC^22,23^ to elucidate the epilepsy brain network. Given the adjacency matrix *A* of a network, α-centrality matrix *C*_*αn*_ is defined as:

$${C}_{\alpha n}=A+ \alpha {A}^{2}+ {\alpha }^{2}{A}^{3}+\dots + {\alpha }^{n}{A}^{n+1}$$

Then it is normalized by divided by the sum of all elements ($\sum {C}_{\alpha n}$), and we have

$${\text{nAC}} = \mathop {\lim }\limits_{{n \to \infty }} \frac{{C_{{\alpha n}} }}{{\sum {C_{{\alpha n}} } }}$$

For the actual calculation, we set *n* = 20, because nAC value converged to a constant value. The nAC enables us to successively assess the local to global network properties by changing the parameter (α) from zero to the reciprocal of the maximum eigenvalue (λ) of the adjacency matrix for each region. The value of nAC at α = 0 (referred to as nAC_0_) corresponds to the degree centrality and that at α = 1/λ (referred to as nAC_1_) corresponds to the eigenvector centrality.

We defined aR_0_ and aR_1_ as the regions whose nAC_0_ and nAC_1_ are larger than the mean + 2 SD of these values at the corresponding region for the age- and sex-matched control data, respectively. Healthy controls were divided into males and females, and control mean and SD at each age were calculated by linear regression for each sex.

### Statistical analysis

We compared the distribution of nAC_0_ and nAC_1_ values for each epilepsy patient with the distributions of age- and sex-matched controls by Chi-square test. Spearman’s correlation analysis was used to check the relationship between the number of aR_0_ or aR_1_ and the number of AEDs, QOLIE-31^54,55^, and LSSS^56^. We used Spearman’s partial correlation analysis with age as nuisance covariate to check the relationship between the number of aR_0_ or aR_1_ and the duration of illness, because the duration of illness was related to the age. The percentage of functionally connected abnormal regions (aR_0_) was calculated as follows: the number of edges among all the aR_0_ regions in each patient divided by the number of possible edges, that is *C*(*n*, 2), where *C* is the combination formula and *n* is the number of aR_0_. The values were used to check the relationship with the clinical parameters by Spearman’s correlation analysis. We calculated the percentage of abnormal regions (aR_0_ and aR_1_) that were within the DMN^25^ (Supplementary Table S2) and the value was compared with the expected percentage calculated by the number of DMN regions (n = 100) divided by the number of all regions (n = 388) using Chi-square test.

We used the kappa statistic to check how different nAC_0_ and nAC_1_ discriminate brain regions to be normal or abnormal using the number of corresponding normal brain regions and corresponding abnormal brain regions.

## Supplementary Information

Supplementary Information

## Data Availability

The data generated during this are available from the corresponding author upon reasonable request.
