# Supplementary material for: Regional abnormality of functional connectivity is associated with clinical manifestations in individuals with intractable focal epilepsy
Source: Sci Rep. 2021 Jan 15;11:1545. doi: 10.1038/s41598-021-81207-6 (PMC7810833; doi:10.1038/s41598-021-81207-6)
Supplement: Supplementary file 1 — Supplementary Information [file 41598_2021_81207_MOESM1_ESM.pdf]

Supplementary materials for:

Regional abnormality of functional connectivity is associated with clinical manifestations in individuals with intractable focal epilepsy

Authors

Yasuo Nakai*[a], Hiroki Nishibayashi[a], Tomohiro Donishi[b], Masaki Terada[c], Naoyuki Nakao[a], Yoshiki Kaneoke[b]

Affiliations

[a]Department of Neurological Surgery, [b]Department of System Neurophysiology, Wakayama Medical University, Wakayama, 641-8509, Japan, [c]Wakayama-Minami Radiology Clinic, Wakayama, 641-0012, Japan

Corresponding Author*: Yasuo Nakai, MD.
Department of Neurological Surgery, Wakayama Medical University
811-1 Kimiidera, Wakayama, 641-8509, Japan.
Phone: +81-73-447-2300; E-mail: ynakai@wakayama-med.ac.jp

Supplementary Table S1: Demographic data of 25 epilepsy patients

Case	Age (years old)	Sex	Duration (years)	Etiology	Seizure type	Seizure frequency	Number of AEDs	Scalp EEG IID/ictal	MRI findings (lesion location)	Type of epilepsy (Estimated focus)	Pathology (if applicable)
1	46	F	42	Structural	Focal, Aware, Motor Onset (tonic)	Daily	3	C4/ diffuse	Focal cortical dysplasia (R supplementary motor area, cingulate cortex)	Focal (R F)	Focal cortical dysplasia (IIb)
2	43	F	5	Structural	Focal, Impaired Awareness Motor Onset (hyperkinetic)	Monthly	1	NS	Focal cortical dysplasia (R rectal gyrus, L frontal gyrus)	Focal (L F)	
3	31	M	18	Structural	Focal, Aware, Motor Onset (myoclonic)	Weekly	4	P4, C4/ non focal	closed-lip schizencephaly (R central sulcus)	Multilobar (R. FT)	Migratory neurons in subcortical region, gliosis
4	19	M	12	Structural	Focal, Impaired Awareness, Motor Onset (tonic)	Daily, nocturnal	4	T3, F7, F3	Polymicrogyria (L temporoparietal cortex)	Focal (L FT)	Dysembryoplastic neuroepithelial tumor
5	51	M	22	Structural	Focal, Impaired awareness, Motor Onset (automatism)	Weekly	1	F7, T3	L temporal tip	TLE (L T)	Epidermoid
6	38	F	18	Structural	Focal Onset, Impaired Awareness, Motor Onset (automatism), focal to bilateral tonic-clonic	Daily	3	F7	L amygdalohippocampus	TLE (L T)	
7	45	F	39	Structural	Focal, Impaired Awareness, Motor Onset (automatism)	Monthly	2	F8	R hippocampus	TLE (R T)	

8	66	F	26	Structural	Focal, Impaired Awareness, Motor Onset (automatism)	Weekly	5	T3, Fp1	L mesial temporal lobe	TLE (L. T)	Low grade glioma
9	45	M	25	Infectious	Focal, Impaired Awareness, Motor Onset (automatism)	Weekly	3	F7, T3	L mesial temporal lobe	TLE (L T)	
10	30	F	20	Structural	Focal, Impaired Awareness, Non-Motor onset (behavior arrest)	Weekly	4	bil.T, P, O/ diffuse	Bil. parietooccipital cortex	Multilobar (Bil. PO)	
11	23	M	1	Structural	Focal, Aware, Motor Onset (tonic, clonic)	Monthly	1	F3, C3	Periventricular nodular heterotopia	Multilobar (L FP	
12	41	M	27	Structural	Focal, Impaired Awareness, Motor Onset (automatism)	Weekly	3	T4, T6 / T4, T6	NS	TLE (R T)	Gliosis
13	16	M	1	Unknown	Focal, Aware, Motor Onset (tonic, clonic)	Weekly	2	C4	NS	Focal (R F)	
14	27	M	6	Unknown	Focal, Aware, Motor Onset (tonic, clonic)	Monthly	2	P3/ non focal	NS	Focal (R P)	
15	37	M	24	Unknown	Focal, Aware, Non-Motor Onset (cognitive)	Weekly	3	T5, T3, O1	NS	Multilobar (L TO)	
16	40	F	37	Unknown	Focal, Aware, Motor Onset (tonic)	Weekly	3	T3, F7/ T5, O1	L hemispheric atrophy	Multilobar (L TO)	
17	36	M	8	Unknown	Focal, Impaired Awareness, Motor Onset (automatism)	Monthly	3	Fp1, F3, T3	NS	Focal (L F)	
18	27	M	23	Unknown	Focal, Impaired Awareness, Motor Onset (automatism)	Weekly	3	Fp1, F7/ F3, T3	NS	Multilobar (L FT)	

19	37	M	25	Unknown	Focal, Impaired Awareness, Motor Onset (atonic)	Monthly	3	F4, F3, Fp2, Fp1/ diffuse	NS	Multilobar (Bil. F)	
20	51	F	41	Unknown	Focal, Aware, Motor Onset (tonic), focal to bilateral tonic-clonic	Daily	3	F8, F4, Fp2, Fp1	NS	Multilobar (Bil. F)	
21	35	M	1	Unknown	Focal, Impaired Awareness, Motor Onset (automatism), focal to bilateral tonic-clonic	Monthly	1	T4	NS	TLE (R T)	
22	43	M	14	Unknown	Focal, Aware, Non-motor onset (behavior arrest)	Weekly	3	F8 / F8	NS	TLE (R T)	
23	70	M	3	Unknown	Focal, Impaired Awareness, Motor Onset (automatism), focal to bilateral tonic-clonic	Monthly	2	F8, T4	NS	TLE (R T)	
24	40	F	17	Unknown	Focal, Impaired awareness, Motor Onset (automatism)	Weekly	3	T3	NS	TLE (L T)	
25	41	M	41	Unknown	Focal, Impaired Awareness, Motor Onset (automatism)	Daily	4	T3, P3, O1	NS	Multilobar (L TPO)	

Abbreviations; TLE: temporal lobe epilepsy, IID: interictal discharge, NS: no significant finding, L: left, R: right, Bil.: bilateral, F: frontal lobe, T: temporal lobe, P: parietal lobe, FT: fronto-parietal lobes, TO: temporo-occipital lobes, FP: fronto-parietal lobes, PO: parieto-occipital lobes, TPO: temporo-parieto-occipital lobes

Supplementary Table S2: **Definition of 388 anatomical regions**

*Regions in DMN. ** No. 1-368 in AICHA, No. 369-388 in AAL

No.	Region name	(Original Index**)
1	G_Frontal_Sup-1-L	(1) *
2	G_Frontal_Sup-1-R	(2) *
3	G_Frontal_Sup-2-L	(3) *
4	G_Frontal_Sup-2-R	(4) *
5	G_Frontal_Sup-3-L	(5) *
6	G_Frontal_Sup-3-R	(6) *
7	S_Sup_Frontal-1-L	(7) *
8	S_Sup_Frontal-1-R	(8) *
9	S_Sup_Frontal-2-L	(9) *
10	S_Sup_Frontal-2-R	(10)*
11	S_Sup_Frontal-3-L	(11)*
12	S_Sup_Frontal-3-R	(12)*
13	S_Sup_Frontal-4-L	(13)*
14	S_Sup_Frontal-4-R	(14)*
15	S_Sup_Frontal-5-L	(15)
16	S_Sup_Frontal-5-R	(16)
17	S_Sup_Frontal-6-L	(17)
18	S_Sup_Frontal-6-R	(18)
19	G_Frontal_Mid-1-L	(19)
20	G_Frontal_Mid-1-R	(20)
21	G_Frontal_Mid-2-L	(21)
22	G_Frontal_Mid-2-R	(22)
23	G_Frontal_Mid-3-L	(23)*
24	G_Frontal_Mid-3-R	(24)
25	G_Frontal_Mid-4-L	(25)*
26	G_Frontal_Mid-4-R	(26)
27	G_Frontal_Mid-5-L	(27)
28	G_Frontal_Mid-5-R	(28)
29	S_Inf_Frontal-1-L	(29)
30	S_Inf_Frontal-1-R	(30)
31	S_Inf_Frontal-2-L	(31)
32	S_Inf_Frontal-2-R	(32)
33	G_Frontal_Inf_Tri-1-L	(33) *
34	G_Frontal_Inf_Tri-1-R	(34) *
35	G_Frontal_Sup_Orb-1-L	(35)
36	G_Frontal_Sup_Orb-1-R	(36)
37	G_Frontal_Mid_Orb-1-L	(37)
38	G_Frontal_Mid_Orb-1-R	(38)
39	G_Frontal_Mid_Orb-2-L	(39)
40	G_Frontal_Mid_Orb-2-R	(40)
41	G_Frontal_Inf_Orb-1-L	(41)
42	G_Frontal_Inf_Orb-1-R	(42)
43	G_Frontal_Inf_Orb-2-L	(43)
44	G_Frontal_Inf_Orb-2-R	(44)
45	S_Orbital-1-L	(45)
46	S_Orbital-1-R	(46)
47	S_Orbital-2-L	(47)
48	S_Orbital-2-R	(48)
49	S_Olfactory-1-L	(49)
50	S_Olfactory-1-R	(50)
51	S_Precentral-1-L	(51)
52	S_Precentral-1-R	(52)
53	S_Precentral-2-L	(53)
54	S_Precentral-2-R	(54)
55	S_Precentral-3-L	(55)
56	S_Precentral-3-R	(56)
57	S_Precentral-4-L	(57)
58	S_Precentral-4-R	(58)
59	S_Precentral-5-L	(59)
60	S_Precentral-5-R	(60)
61	S_Precentral-6-L	(61)
62	S_Precentral-6-R	(62)
63	S_Rolando-1-L	(63)
64	S_Rolando-1-R	(64)
65	S_Rolando-2-L	(65)
66	S_Rolando-2-R	(66)
67	S_Rolando-3-L	(67)
68	S_Rolando-3-R	(68)
69	S_Rolando-4-L	(69)
70	S_Rolando-4-R	(70)
71	S_Postcentral-1-L	(71)
72	S_Postcentral-1-R	(72)
73	S_Postcentral-2-L	(73)
74	S_Postcentral-2-R	(74)
75	S_Postcentral-3-L	(75)
76	S_Postcentral-3-R	(76)
77	G_Parietal_Sup-1-L	(77)
78	G_Parietal_Sup-1-R	(78)
79	G_Parietal_Sup-2-L	(79)

80 G_Parietal_Sup-2-R (80)
81 G_Parietal_Sup-3-L (81)
82 G_Parietal_Sup-3-R (82)
83 G_Parietal_Sup-4-L (83)
84 G_Parietal_Sup-4-R (84)
85 G_Parietal_Sup-5-L (85)
86 G_Parietal_Sup-5-R (86)
87 G_Supramarginal-1-L (87)
88 G_Supramarginal-1-R (88)
89 G_SupraMarginal-2-L (89)
90 G_SupraMarginal-2-R (90)
91 G_Supramarginal-3-L (91)
92 G_Supramarginal-3-R (92)
93 G_Supramarginal-4-L (93)
94 G_Supramarginal-4-R (94)
95 G_SupraMarginal-5-L (95)
96 G_SupraMarginal-5-R (96)
97 G_SupraMarginal-6-L (97)
98 G_SupraMarginal-6-R (98)
99 G_SupraMarginal-7-L (99) *
100 G_SupraMarginal-7-R (100) *
101 G_Angular-1-L (101) *
102 G_Angular-1-R (102) *
103 G_Angular-2-L (103) *
104 G_Angular-2-R (104) *
105 G_Angular-3-L (105) *
106 G_Angular-3-R (106) *
107 G_Parietal_Inf-1-L (107) *
108 G_Parietal_Inf-1-R (108) *
109 S_Intraparietal-1-L (109) *
110 S_Intraparietal-1-R (110) *
111 S_Intraparietal-2-L (111) *
112 S_Intraparietal-2-R (112) *
113 S_Intraparietal-3-L (113) *
114 S_Intraparietal-3-R (114) *
115 S_Intraoccipital_G_Occipital_Mid-1-L (115, 133)
116 S_Intraoccipital-G_Occipital_Mid-1-R (116, 134)
117 G_Occipital_Pole-1-L (117)
118 G_Occipital_Pole-1-R (118)
119 G_Occipital_Lat-1-L (119)
120 G_Occipital_Lat-1-R (120)
121 G_Occipital_Lat-2-L (121)
122 G_Occipital_Lat-2-R (122)
123 G_Occipital_Lat-3-L (123)
124 G_Occipital_Lat-3-R (124)
125 G_Occipital_Lat-4-L (125)
126 G_Occipital_Lat-4-R (126)
127 G_Occipital_Lat-5-L (127)
128 G_Occipital_Lat-5-R (128)
129 G_Occipital_Sup-1-L (129)
130 G_Occipital_Sup-1-R (130)
131 G_Occipital_Sup-2-L (131)
132 G_Occipital_Sup-2-R (132)
133 G_Occipital_Mid-2-L (135)
134 G_Occipital_Mid-2-R (136)
135 G_Occipital_Mid-3-L (137)
136 G_Occipital_Mid-3-R (138)
137 G_Occipital_Mid-4-L (139) *
138 G_Occipital_Mid-4-R (140) *
139 G_Occipital_Inf-1-L (141)
140 G_Occipital_Inf-1-R (142)
141 G_Occipital_Inf-2-L (143)
142 G_Occipital_Inf-2-R (144)
143 G_Insula-anterior-1-L (145)
144 G_Insula-anterior-1-R (146)
145 G_Insula-anterior-2-L (147)
146 G_Insula-anterior-2-R (148)
147 G_Insula-anterior-3-L (149)
148 G_Insula-anterior-3-R (150)
149 G_Insula-anterior-4-L (151)
150 G_Insula-anterior-4-R (152)
151 G_Insula-anterior-5-L (153)
152 G_Insula-anterior-5-R (154)
153 G_Insula-posterior-1-L (155)
154 G_Insula-posterior-1-R (156)
155 G_Rolandic_Oper-1-L (157)
156 G_Rolandic_Oper-1-R (158)
157 G_Rolandic_Oper-2-L (159)
158 G_Rolandic_Oper-2-R (160)
159 G_Temporal_Sup-1-L (161)
160 G_Temporal_Sup-1-R (162)
161 G_Temporal_Sup-2-L (163)
162 G_Temporal_Sup-2-R (164)
163 G_Temporal_Sup-3-L (165)
164 G_Temporal_Sup-3-R (166)
165 G_Temporal_Sup-4-L (167)
166 G_Temporal_Sup-4-R (168)
167 S_Sup_Temporal-1-L (169) *
168 S_Sup_Temporal-1-R (170) *
169 S_Sup_Temporal-2-L (171) *
170 S_Sup_Temporal-2-R (172) *
171 S_Sup_Temporal-3-L (173)
172 S_Sup_Temporal-3-R (174)
173 S_Sup_Temporal-4-L (175)
174 S_Sup_Temporal-4-R (176)
175 S_Sup_Temporal-5-L (177) *
176 S_Sup_Temporal-5-R (178) *
177 G_Temporal_Mid-1-L (179) *
178 G_Temporal_Mid-1-R (180) *
179 G_Temporal_Mid-2-L (181)
180 G_Temporal_Mid-2-R (182)
181 G_Temporal_Mid-3-L (183) *
182 G_Temporal_Mid-3-R (184) *
183 G_Temporal_Mid-4-L (185)
184 G_Temporal_Mid-4-R (186)
185 G_Temporal_Inf-1-L (187)
186 G_Temporal_Inf-1-R (188)
187 G_Temporal_Inf-2-L (189)
188 G_Temporal_Inf-2-R (190)
189 G_Temporal_Inf-3-L (191)
190 G_Temporal_Inf-3-R (192)
191 G_Temporal_Inf-4-L (193)
192 G_Temporal_Inf-4-R (194)
193 G_Temporal_Inf-5-L (195)

194 G_Temporal_Inf-5-R (196)
195 G_Temporal_Pole_Sup-1-L (197) *
196 G_Temporal_Pole_Sup-1-R (198) *
197 G_Temporal_Pole_Sup-2-L (199) *
198 G_Temporal_Pole_Sup-2-R (200) *
199 G_Temporal_Pole_Mid-1-L (201)
200 G_Temporal_Pole_Mid-1-R (202)
201 G_Temporal_Pole_Mid-2-L (203)
202 G_Temporal_Pole_Mid-2-R (204)
203 G_Temporal_Pole_Mid-3-L (205)
204 G_Temporal_Pole_Mid-3-R (206)
205 G_Frontal_Sup_Medial-1-L (207) *
206 G_Frontal_Sup_Medial-1-R (208) *
207 G_Frontal_Sup_Medial-2-L (209) *
208 G_Frontal_Sup_Medial-2-R (210) *
209 G_Frontal_Sup_Medial-3-L (211) *
210 G_Frontal_Sup_Medial-3-R (212) *
211 S_Anterior_Rostral-1-L (213) *
212 S_Anterior_Rostral-1-R (214) *
213 G_Frontal_Med_Orb-1-L (215) *
214 G_Frontal_Med_Orb-1-R (216) *
215 G_Frontal_Med_Orb-2-L (217)
216 G_Frontal_Med_Orb-2- (218)
217 G_subcallosal-1-L (219)
218 G_subcallosal-1-R (220)
219 G_Supp_Motor_Area-1-L (221)
220 G_Supp_Motor_Area-1-R (222)
221 G_Supp_Motor_Area-2-L (223)
222 G_Supp_Motor_Area-2-R (224)
223 G_Supp_Motor_Area-3-L (225)
224 G_Supp_Motor_Area-3-R (226)
225 S_Cingulate-1-L (227) *
226 S_Cingulate-1-R (228) *
227 S_Cingulate-2-L (229)
228 S_Cingulate-2-R (230)
229 S_Cingulate-3-L (231)
230 S_Cingulate-3-R (232)
231 S_Cingulate-4-L (233)
232 S_Cingulate-4-R (234)
233 S_Cingulate-5-L (235)
234 S_Cingulate-5-R (236)
235 S_Cingulate-6-L (237) *
236 S_Cingulate-6-R (238) *
237 S_Cingulate-7-L (239) *
238 S_Cingulate-7-R (240) *
239 G_Cingulum_Ant-1-L(241) *
240 G_Cingulum_Ant-1-R (242) *
241 G_Cingulum_Ant-2-L(243) *
242 G_Cingulum_Ant-2-R (244) *
243 G_Cingulum_Mid-1_2-L (245, 247)
244 G_Cingulum_Mid-1_2-R (246, 248)
245 G_Cingulum_Mid-3-L (249)
246 G_Cingulum_Mid-3-R (250)
247 G_Cingulum_Post-1-L (251) *
248 G_Cingulum_Post-1-R (252) *
249 G_Cingulum_Post-2_3-L (253, 255) *
250 G_Cingulum_Post-2_3-R (254, 256) *
251 G_Paracentral_Lobule-1-L (257)
252 G_Paracentral_Lobule-1-R (258)
253 G_Paracentral_Lobule-2-L (259)
254 G_Paracentral_Lobule-2-R (260)
255 G_Paracentral_Lobule-3-L (261)
256 G_Paracentral_Lobule-3-R (262)
257 G_Paracentral_Lobule-4-L (263)
258 G_Paracentral_Lobule-4-R (264)
259 G_Precuneus-1-L (265) *
260 G_Precuneus-1-R (266) *
261 G_Precuneus-2-L (267) *
262 G_Precuneus-2-R (268) *
263 G_Precuneus-3-L (269) *
264 G_Precuneus-3-R (270) *
265 G_Precuneus-4-L (271) *
266 G_Precuneus-4-R (272) *
267 G_Precuneus-5-L (273) *
268 G_Precuneus-5-R (274) *
269 G_Precuneus-6-L (275) *
270 G_Precuneus-6-R (276) *
271 G_Precuneus-7-L (277) *
272 G_Precuneus-7-R (278) *
273 G_Precuneus-8-L (279) *
274 G_Precuneus-8-R (280) *
275 G_Precuneus-9-L (281) *
276 G_Precuneus-9-R (282) *
277 S_Parietooccipital-1-L (283)
278 S_Parietooccipital-1-R (284)
279 S_Parietooccipital-2-L (285)
280 S_Parietooccipital-2-R (286)
281 S_Parietooccipital-3-L (287)
282 S_Parietooccipital-3-R (288)
283 S_Parietooccipital-4-L (289)
284 S_Parietooccipital-4-R (290)
285 S_Parietooccipital-5-L (291)
286 S_Parietooccipital-5-R (292)
287 S_Parietooccipital-6-L (293)
288 S_Parietooccipital-6-R (294)
289 G_Cuneus-1-L (295)
290 G_Cuneus-1-R (296)
291 G_Cuneus-2-L (297)
292 G_Cuneus-2-R (298)
293 G_Calcarine-1-L (299)
294 G_Calcarine-1-R (300)
295 G_Calcarine-2-L (301)
296 G_Calcarine-2-R (302)
297 G_Calcarine-3-L (303)
298 G_Calcarine-3-R (304)
299 G_Lingual-1-L (305) *
300 G_Lingual-1-R (306) *
301 G_Lingual-2-L (307)
302 G_Lingual-2-R (308)
303 G_Lingual-3-L (309)
304 G_Lingual-3-R (310)
305 G_Lingual-4-L (311)
306 G_Lingual-4-R (312)
307 G_Lingual-5-L (313)
308 G_Lingual-5-R (314)
309 G_Lingual-6-L (315)

310 G_Lingual-6-R (316)
311 G_Hippocampus-1-L (317)
312 G_Hippocampus-1-R (318)
313 G_Hippocampus-2-L (319)
314 G_Hippocampus-2-R (320)
315 G_ParaHippocampal-1_2_3-L (321, 323, 325) *
316 G_ParaHippocampal-1_2_3-R (322, 324, 326) *
317 G_ParaHippocampal-4-L (327) *
318 G_ParaHippocampal-4-R (328) *
319 G_ParaHippocampal-5-L (329) *
320 G_ParaHippocampal-5-R (330) *
321 G_Fusiform-1-L (331)
322 G_Fusiform-1-R (332)
323 G_Fusiform-2-L (333)
324 G_Fusiform-2-R (334)
325 G_Fusiform-3-L (335)
326 G_Fusiform-3-R (336)
327 G_Fusiform-4_5-L (337, 339)
328 G_Fusiform-4_5-R (338, 340)
329 G_Fusiform-6-L (341)
330 G_Fusiform-6-R (342)
331 G_Fusiform-7-L (343)
332 G_Fusiform-7-R (344)
333 N_Amygdala-1-L (345)
334 N_Amygdala-1-R (346)
335 N_Caudate-1_2-L (347, 349)
336 N_Caudate-1_2-R (348, 350)
337 N_Caudate-3-L (351)
338 N_Caudate-3-R (352)
339 N_Caudate-4-L (353)
340 N_Caudate-4-R (354)
341 N_Caudate-5-L (355)
342 N_Caudate-5-R (356)
343 N_Caudate-6_7-L (357, 359)
344 N_Caudate-6_7-R (358, 360)
345 N_Pallidum-1-L (361)
346 N_Pallidum-1-R (362)
347 N_Putamen-2-L (363)
348 N_Putamen-2-R (364)
349 N_Putamen-3-L (365)
350 N_Putamen-3-R (366)
351 N_Thalamus-1-L (367)
352 N_Thalamus-1-R (368)
353 N_Thalamus-2-L (369)
354 N_Thalamus-2-R (370)
355 N_Thalamus-3-L (371)
356 N_Thalamus-3-R (372)
357 N_Thalamus-4-L (373)
358 N_Thalamus-4-R (374)
359 N_Thalamus-5-L (375)
360 N_Thalamus-5-R (376)
361 N_Thalamus-6-L (377)
362 N_Thalamus-6-R (378)
363 N_Thalamus-7-L (379)
364 N_Thalamus-7-R (380)
365 N_Thalamus-8-L (381)
366 N_Thalamus-8-R (382)
367 N_Thalamus-9-L (383)
368 N_Thalamus-9-R (384)
369 Cerebelum_Crus1_L (9001)
370 Cerebelum_Crus1_R (9002)
371 Cerebelum_Crus2_L (9011)
372 Cerebelum_Crus2_R (9012)
373 Cerebelum_3_L (9021)
374 Cerebelum_3_R (9022)
375 Cerebelum_4_5_L (9031)
376 Cerebelum_4_5_R (9032)
377 Cerebelum_6_L (9041)
378 Cerebelum_6_R (9042)
379 Cerebelum_7b_L (9051)
380 Cerebelum_7b_R (9052)
381 Cerebelum_8_9_10_L (9061, 9071, 9081)
382 Cerebelum_8_9_10_R (9062, 9072, 9082)
383 Vermis_1_2_3 (9100, 9110)
384 Vermis_4_5 (9120)
385 Vermis_6 (9130)
386 Vermis_7 (9140)
387 Vermis_8 (9150)
388 Vermis_9_10 (9160, 9170)

Supplementary Table S3: **Percentage of abnormal regions within the DMN, and Chi-square test for comparing it with the expected value for 25 epilepsy patients**

	aR0			aR1		
Case	(%)	chi2_value	p-value	(%)	chi2_value	p-value
1	1/14 (7.1)	-	-	3/11 (27.3)	0.013307	0.90816
2	8/19 (42.1)	2.7856	0.095118	11/32 (34.4)	1.3489	0.24547
3	10/37 (27.0)	0.033611	0.85454	0/17 (0.0)	-	-
4	**12/21 (57.1)**	**11.42**	**0.000726**	**14/23 (60.9)**	**15.742**	**7.26E-05**
5	17/54 (31.5)	1.0685	0.30129	7/46 (15.2)	-	-
6	5/20 (25.0)	-	-	0/4 (0.0)	-	-
7	**18/21 (85.7)**	**41.697**	**1.07E-10**	**41/56 (73.2)**	**76.995**	**1.71E-18**
8	**12/29 (41.4)**	**3.9902**	**0.045765**	13/40 (32.5)	1.0549	0.30439
9	5/17 (29.4)	0.12304	0.72576	5/18 (27.8)	0.039648	0.84217
10	2/22 (9.1)	-	-	0/3 (0.0)	-	-
11	**5/8 (62.5)**	**5.7594**	**0.016401**	**2/2 (100.0)**	**5.7898**	**0.016119**
12	3/16 (18.8)	-	-	0/27 (0.0)	-	-
13	0/11 (0.0)	-	-	0/2 (0.0)	-	-
14	6/18 (33.3)	0.56394	0.45268	**9/18 (50.0)**	**5.7912**	**0.016107**
15	**19/33 (57.6)**	**19.068**	**1.26E-05**	**26/41 (63.4)**	**33.954**	**5.64E-09**
16	**33/58 (56.9)**	**34.529**	**4.2E-09**	**43/68 (63.2)**	**60.485**	**7.42E-15**
17	3/15 (20.0)	-	-	3/30 (10.0)	-	-
18	7/20 (35.0)	0.9384	0.33269	1/11 (9.1)	-	-
19	8/30 (26.7)	0.013568	0.90727	14/37 (37.8)	3.1119	0.077722
20	13/35 (37.1)	2.5995	0.1069	**25/37(67.6)**	**37.345**	**9.9E-10**
21	5/10 (50.0)	3.1492	0.075963	4/8 (50.0)	2.5061	0.11341
22	1/5 (20.0)	-	-	0/2 (0.0)	-	-
23	0/12 (0.0)	-	-	0/5 (0.0)	-	-
24	4/20 (20.0)	-	-	8/37 (21.6)	-	-
25	8/35 (22.9)	-	-	7/26 (26.9)	0.019261	0.88962

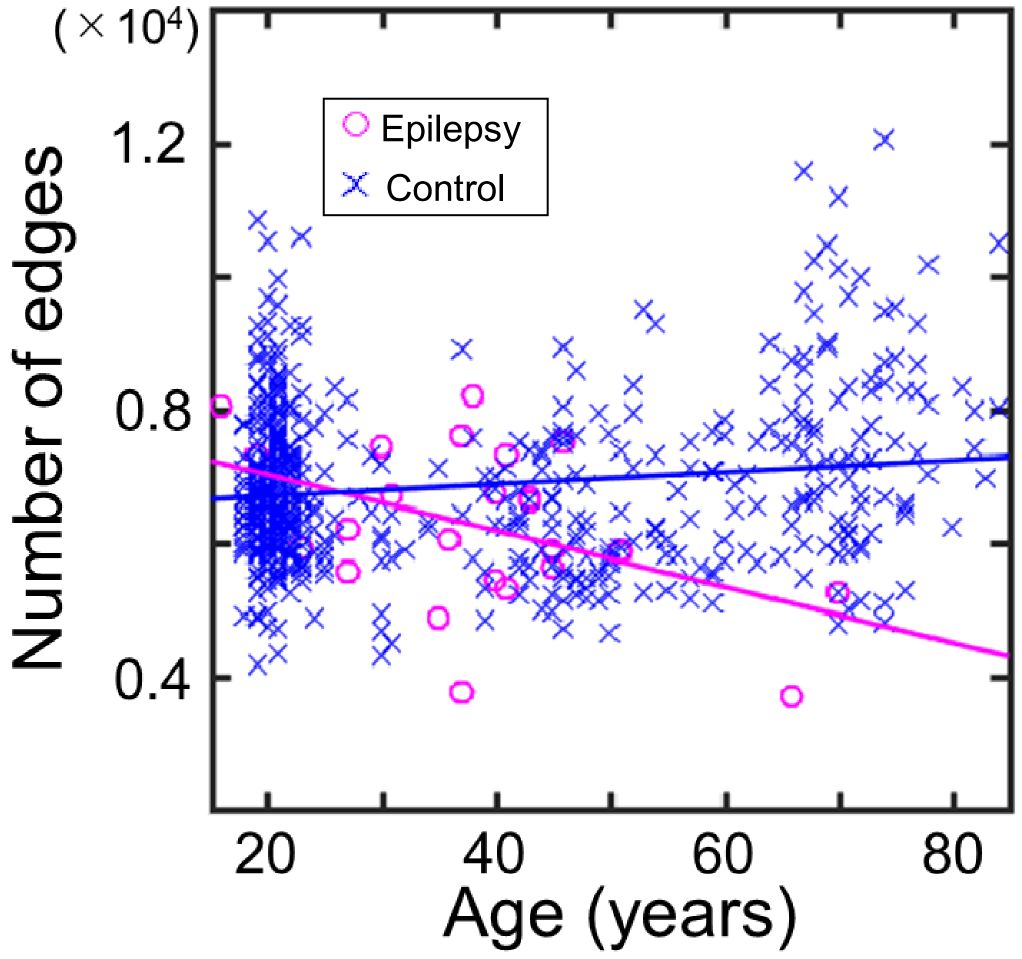

Supplementary Figure S1: Distributions of mean nAC values and the number of edges for each group.

The number of edges was significantly different between controls and epilepsy patients (df = 1, F = 7.19, p = 0.0075, ANCOVA with age as a nuisance covariate). There was also significant interaction between group and age (df =1, F = 6.97, p = 0.0085).

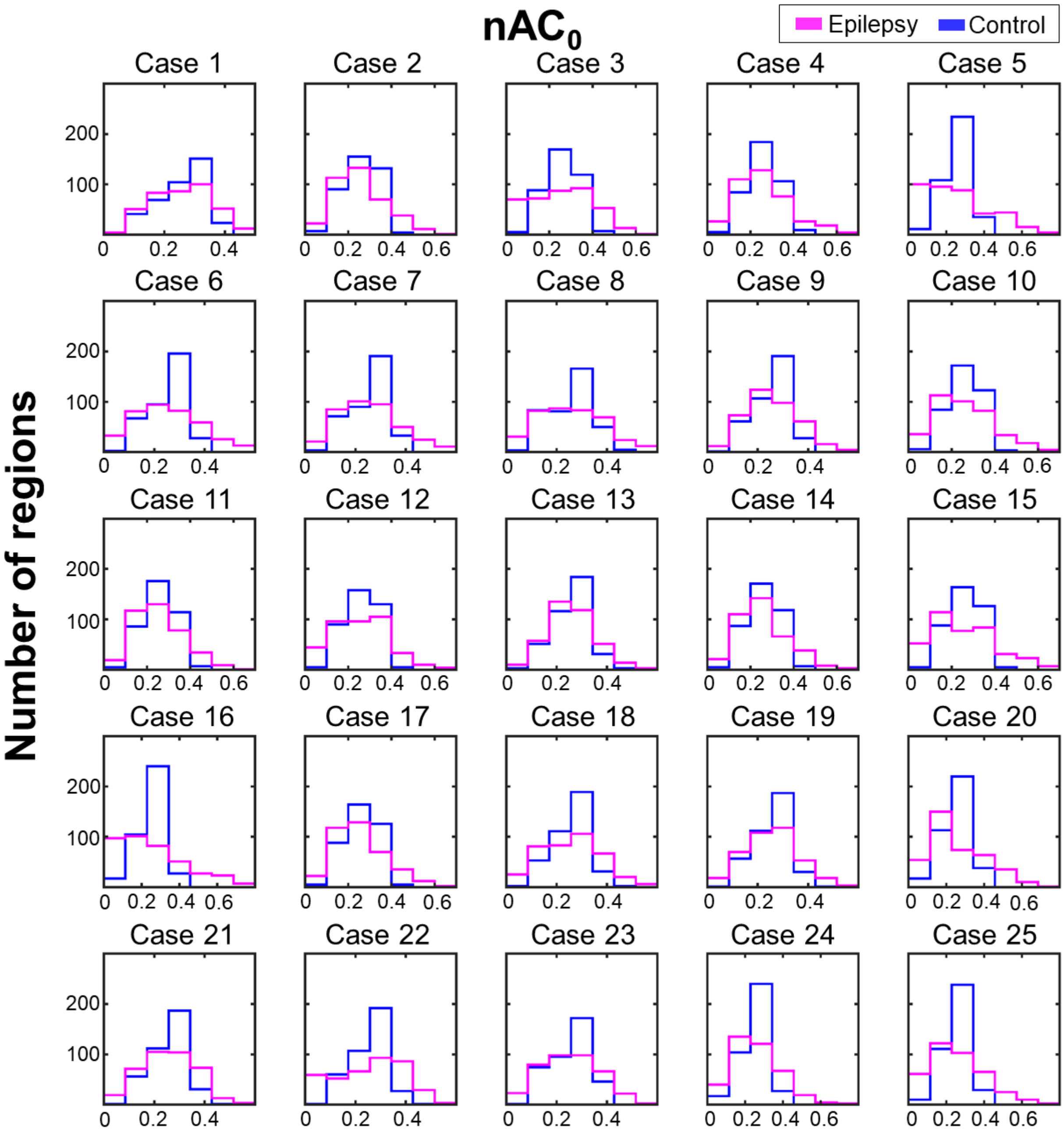

Supplementary Figure S2: The distributions of nAC$_0$ for all cases

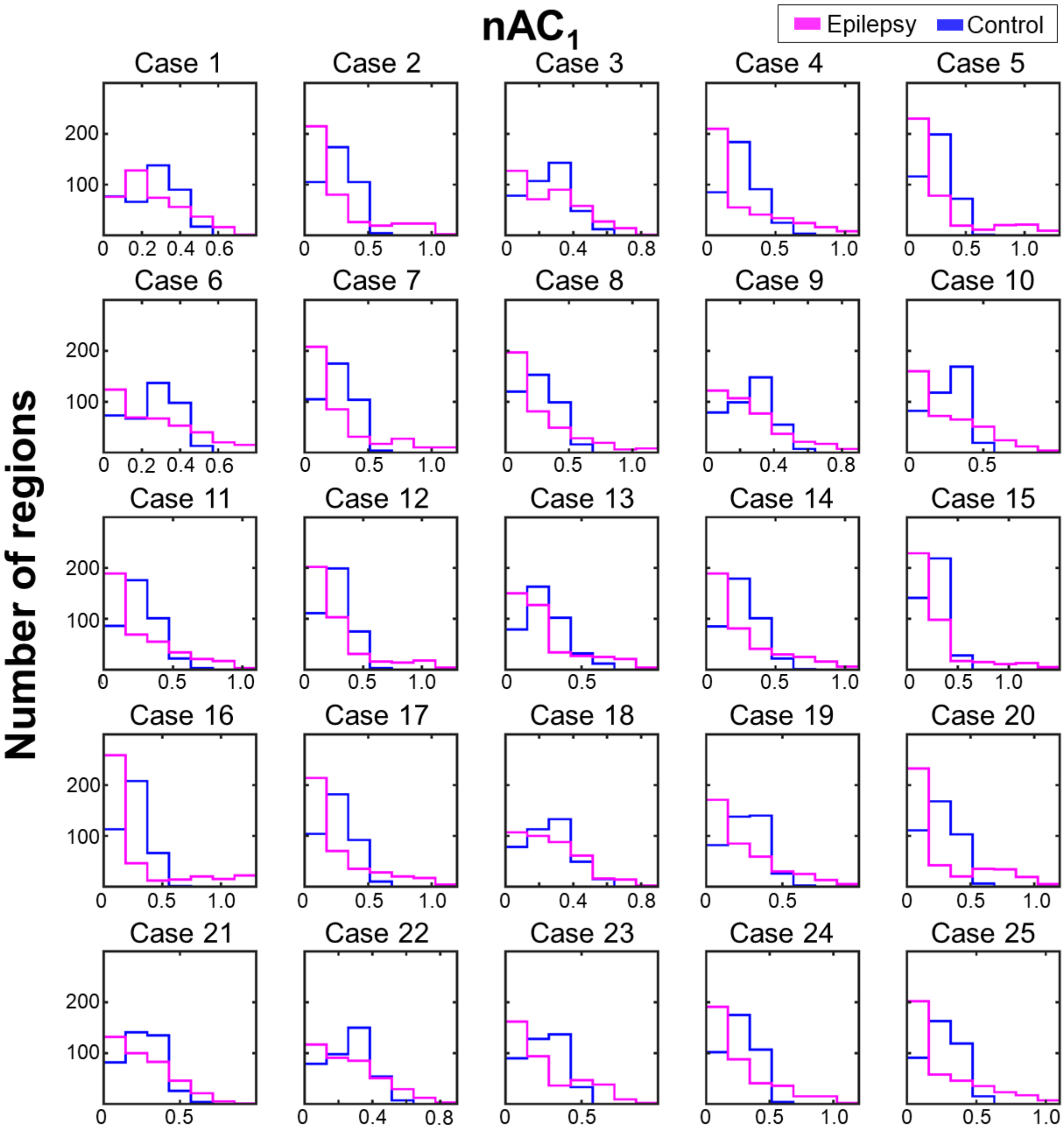

Supplementary Figure S3: The distributions of nAC$_1$ for all cases

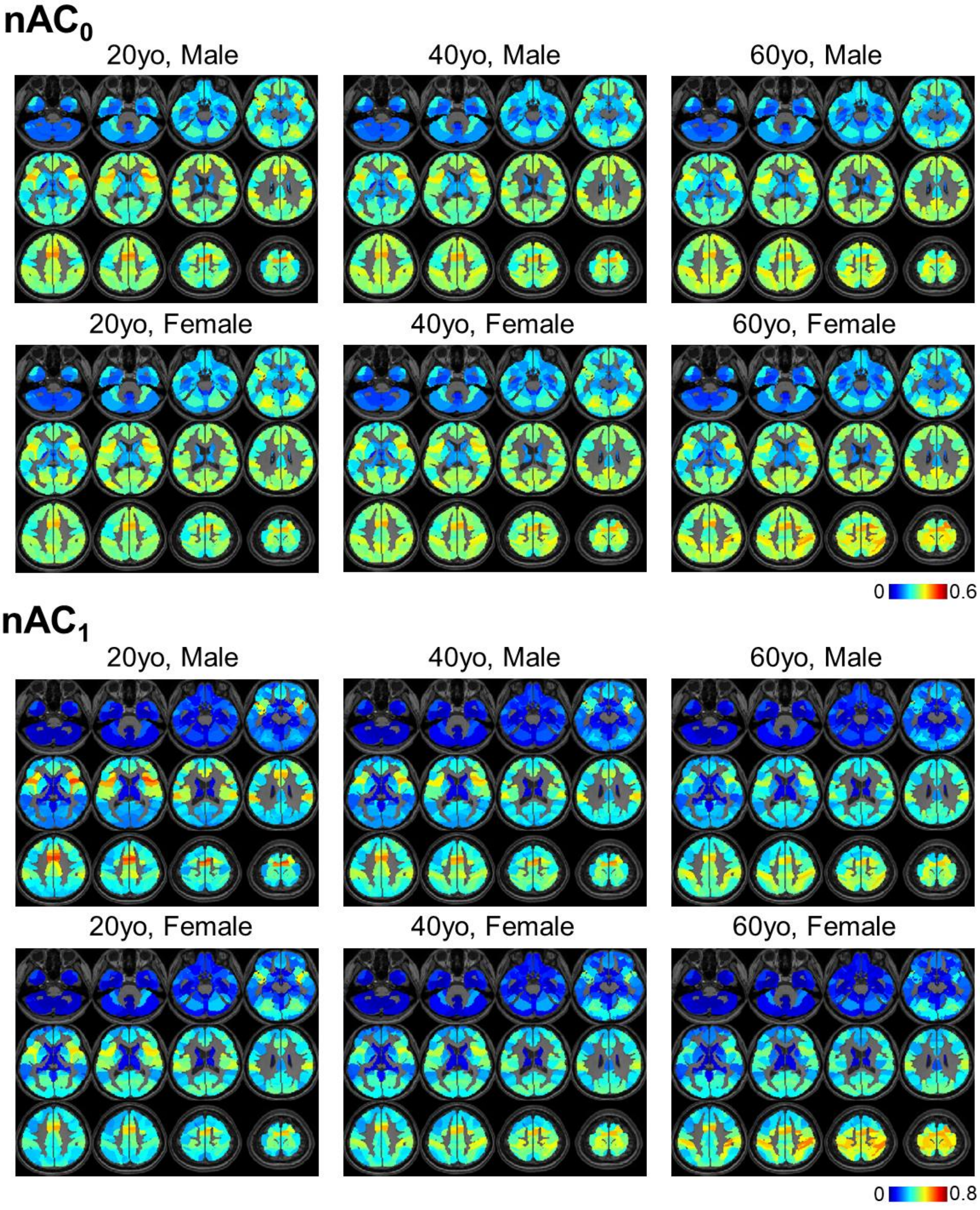

Supplementary Figure S4: The spatial distributions of nAC_0 and nAC_1 for 20, 40, and 60 years old for males and females.

The values for each age were calculated by the linear regression of the healthy control data for each sex.

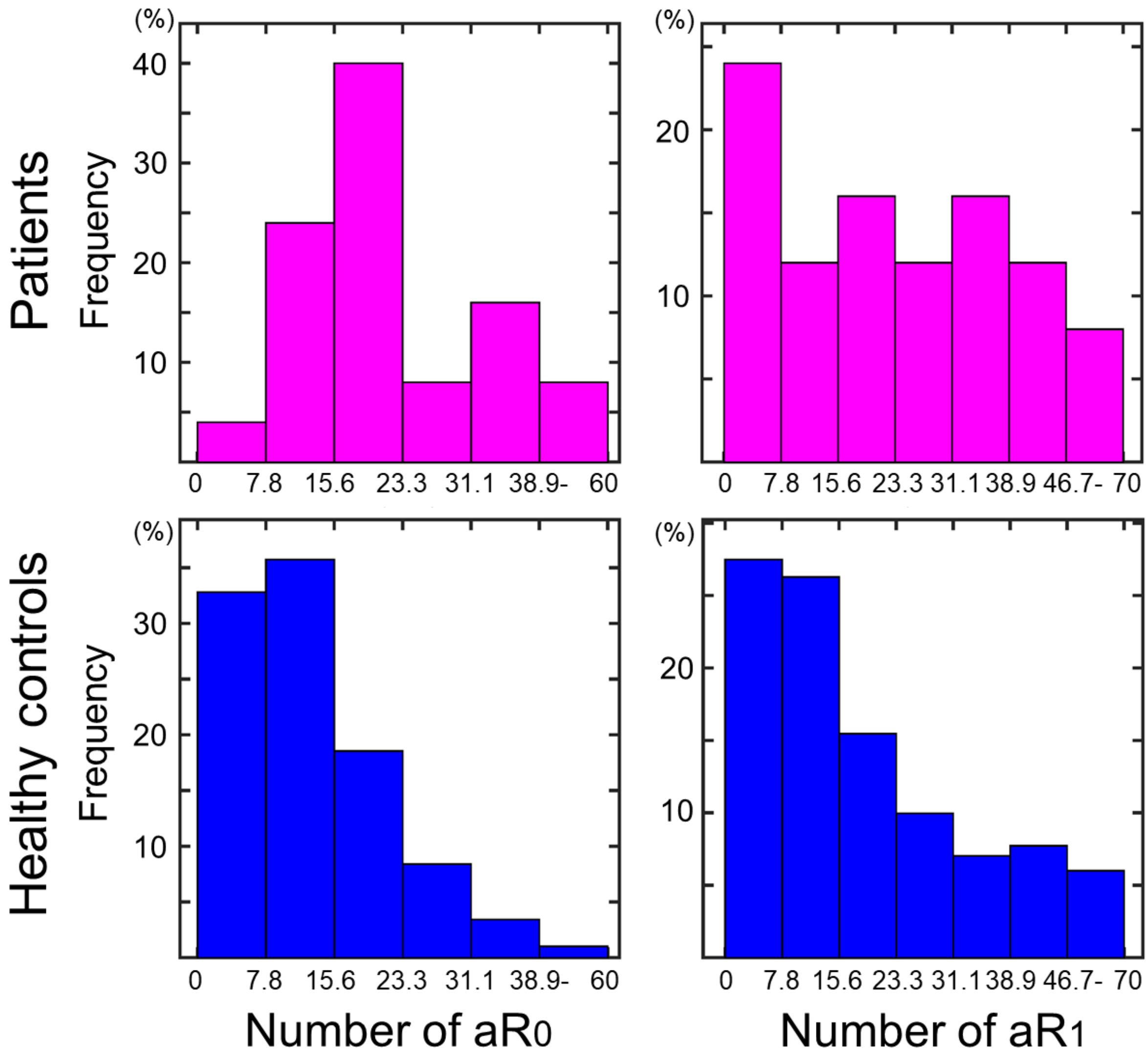

Supplementary Figure S5: The relative frequency distributions of the number of R_0 and aR_1 for epilepsy patients and healthy controls.

Chi-square tests revealed that distributions for the patients were significantly different from those for control data ($\chi^2 = 147$, $p < 0.00001$ for aR_0; $\chi^2 = 23.04$, $p = 0.00078$ for aR_1).

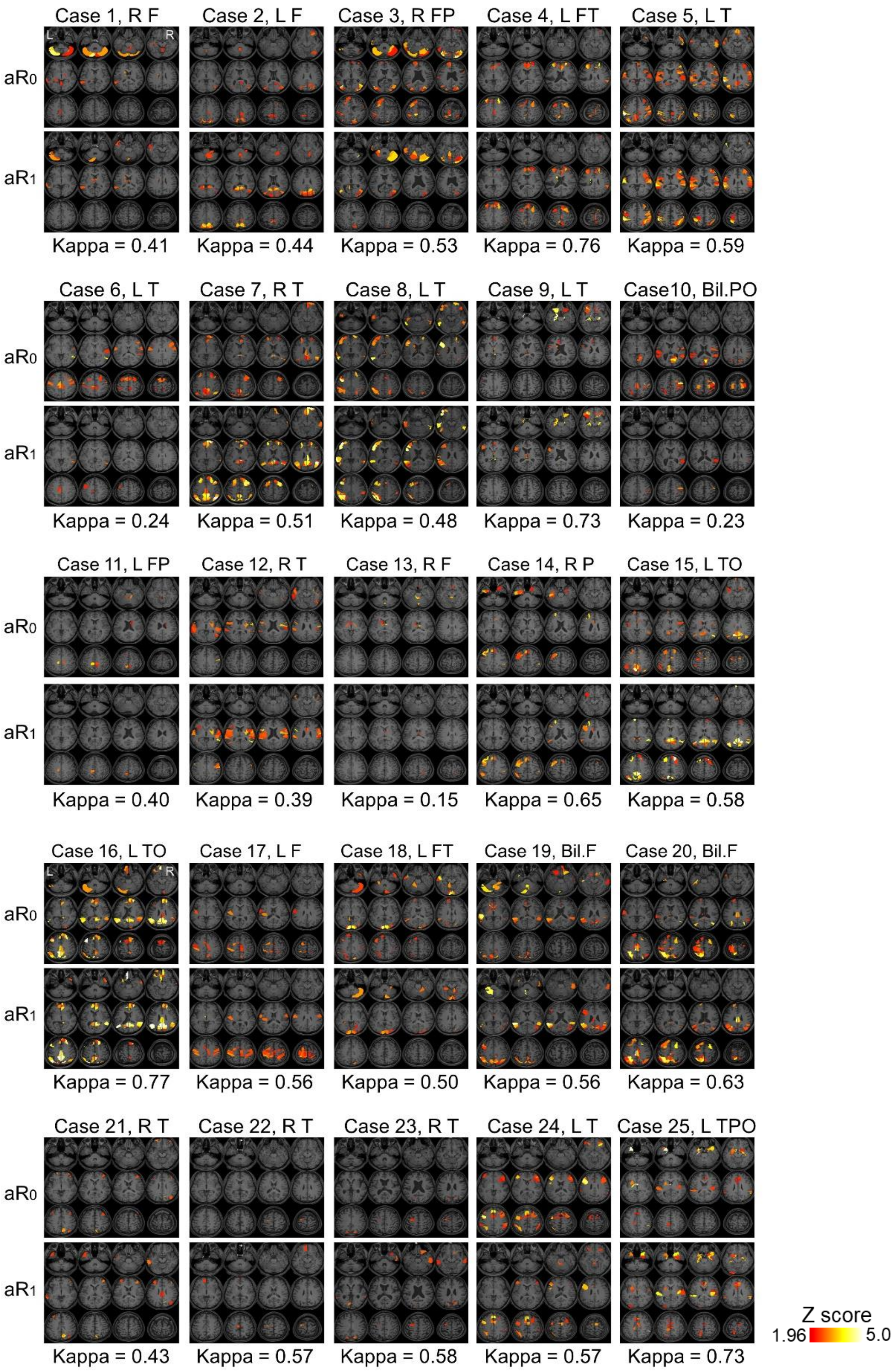

Supplementary Figure S6: The spatial distributions of aR_0 and aR_1 and values of kappa statistic for all cases.

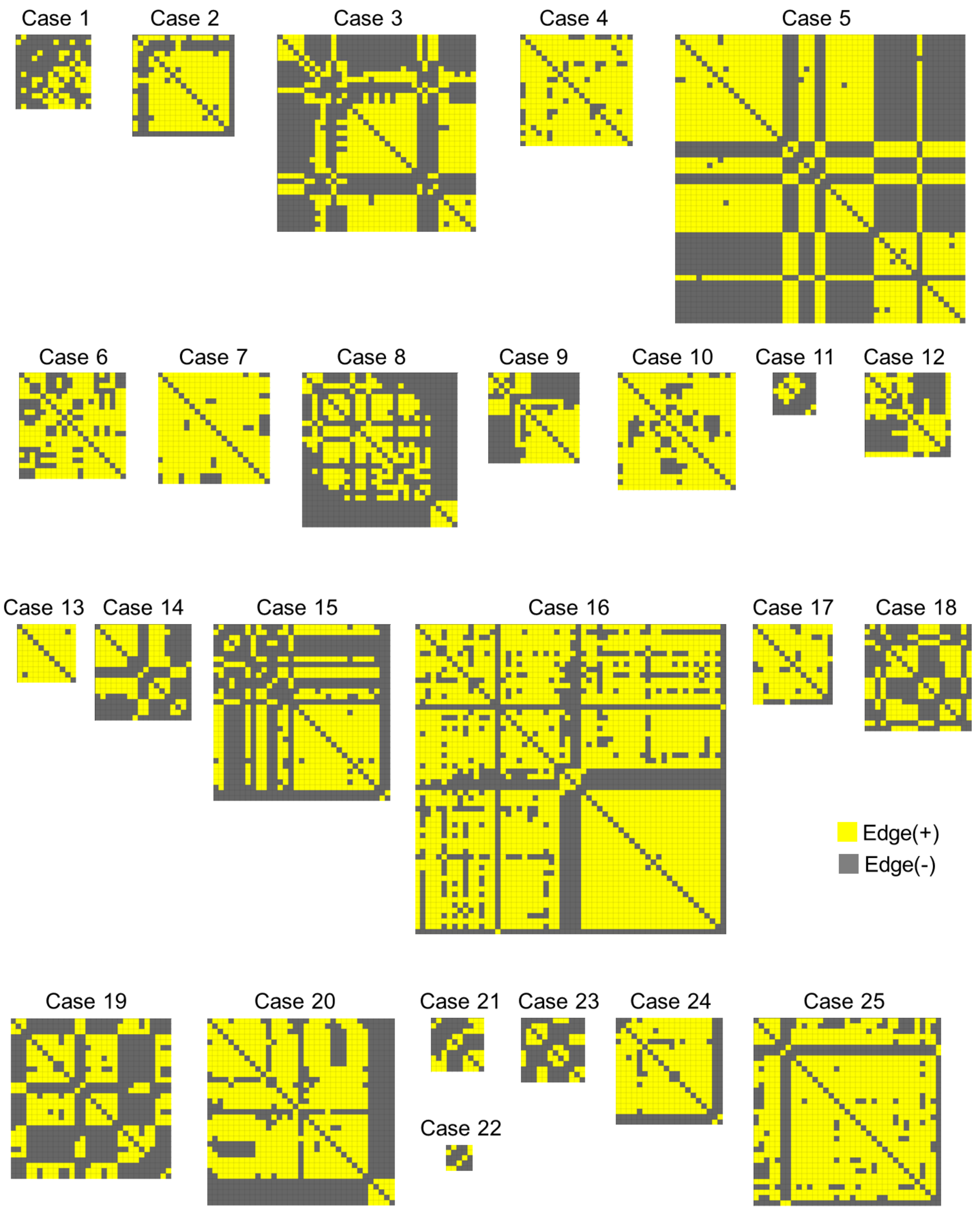

Supplementary Figure S7: Visual matrix of the functional connectivity (edge) between each abnormal region (aR_0) for all cases.
